# Global analyses of mRNA translational control during early *Drosophila *embryogenesis

**DOI:** 10.1186/gb-2007-8-4-r63

**Published:** 2007-04-22

**Authors:** Xiaoli Qin, Soyeon Ahn, Terence P Speed, Gerald M Rubin

**Affiliations:** 1 Howard Hughes Medical Institute, Department of Molecular & Cellular Biology, University of California, Berkeley, Berkeley CA 94720, USA; 2 InterMune, Inc., Brisbane, CA 94005, USA; 3 Department of Statistics, University of California, Berkeley, Berkeley CA 94720, USA; 4 Janelia Farm Research Campus, Howard Hughes Medical Institutes,19700 Helix Drive, Ashburn, VA 20147

## Abstract

**Background:**

In many animals, the first few hours of life proceed with little or no transcription, and developmental regulation at these early stages is dependent on maternal cytoplasm rather than the zygotic nucleus. Translational control is critical for early *Drosophila *embryogenesis and is exerted mainly at the gene level. To understand post-transcriptional regulation during *Drosophila *early embryonic development, we used sucrose polysomal gradient analyses and GeneChip analysis to illustrate the translation profile of individual mRNAs.

**Results:**

We determined ribosomal density and ribosomal occupancy of over 10,000 transcripts during the first ten hours after egg laying.

**Conclusion:**

We report the extent and general nature of gene regulation at the translational level during early *Drosophila *embryogenesis on a genome-wide basis. The diversity of the translation profiles indicates multiple mechanisms modulating transcript-specific translation. Cluster analyses suggest that the genes involved in some biological processes are co-regulated at the translational level at certain developmental stages.

## Abstract 2

The polysomal profiles of over 15,000 transcripts during the first ten hours after egg laying have been determined.

## Background

In many animal species, the first few hours of life proceed with little or no transcription, and regulation of developmental events at these early stages is conferred by maternal cytoplasm rather than transcriptional activity in the zygotic nucleus. During the first two hours after fertilization, *Drosophila *embryos undergo 13 zygotic division cycles (Bownes' stages 1-4) and are syncytial in that the nuclei divide in a common cytoplasm without cytokenesis, except that pole cells, precursors to germline, are segregated in cycle 10. Synthesis of rRNA, tRNAs, 5S RNAs, snRNAs, poly(A)+ RNAs, and histone mRNAs is not detectable until cycle 11 or 12. Both spatial control and temporal control of mRNA translation are implemented in the early patterning of the *Drosophila *embryo. The basic embryonic body plan, defined by both anterior-posterior and dorso-ventral axes, as well as precursors for terminal structures, relies on the regulation of mRNA localization and coupled regulation of mRNA translation. Complete inhibition of protein synthesis with translation inhibitors, for example, cycloheximide, puromycin or pactamycin, quickly and entirely blocks mitotic cycles and arrests development [1,2]. After zygotic transcription begins at mitotic cycle 13 (about 1.5-2 hours after fertilization), the efficient use of zygotic transcripts depends on the degradation of maternal mRNA after fertilization [3].

The modulation of translation can be exerted by both general mechanisms that influence the mRNA population as a whole and selective mechanisms that influence individual mRNAs or small groups of mRNAs. In *Drosophila*, multiple mechanisms of translational control have been previously reported, such as control by RNA degradation, transcript localization and polyadenylation. Cis-regulatory RNA elements are generally found within the 5' or 3' untranslated regions of mRNAs (5' UTRs and 3' UTRs). For example, specific sequence elements in the 3' UTRs of *Drosophila bicoid *and *nanos *mRNAs guide these mRNAs to the anterior and posterior poles of the developing embryo, respectively. Unlocalized *bicoid *or *nanos *mRNAs are bound to translational repressor molecules, and proper localization of both mRNAs relieves the repression and permits their translation [4-8].

The sedimentation of a given mRNA when a cell extract is applied to a sucrose density gradient is determined by the number of its associated ribosomes. Changes in the size (the number of ribosomes per mRNA) and the amount (amplitude) of a specific polysome-associated transcript in a gradient can indicate regulation of protein synthesis [9]. Comparison of polysomal associated mRNA between developmental stages using microarray analysis provides an approach to a genomic-wide investigation of translation dynamics during development. This method has been successful in identifying cellular internal ribosomal entry sites (IRES) that are translated in mitotic HeLa cells and to describe the global translation profile of *Saccharomyces cerevisiae *[10-12].

We have taken a similar approach to a genome-level investigation of translational regulation during early embryogenesis in *Drosophila*. In this study, we have fractionated embryo extracts from a series of early stages by sedimentation on sucrose density gradients and analyzed the RNA components of these fractions using the *Drosophila *GeneChip Genome 2.0 array (Affymetrix, Santa Clara). Our analysis has focused on analyzing ribosomal density, generally and for individual transcripts, global translational activity during the first 10 hours after egg laying and coordination between transcription and translation regulation.

## Results and discussion

**Figure 1:**
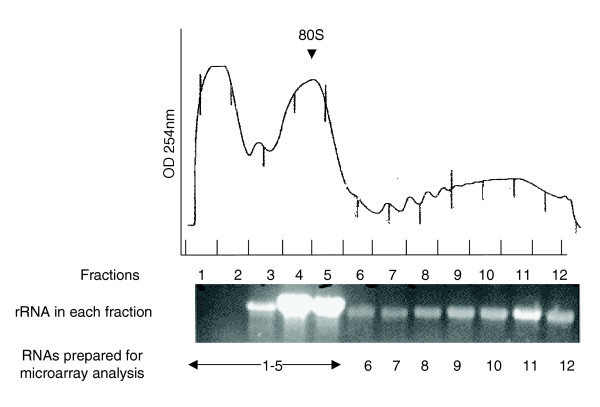
Representative ultraviolet absorbance profile for embryonic RNA fractionated by 20% to 50% sucrose density gradients. The 80S ribosomal subunit position is indicated. RNAs were purified from each fraction, electrophoresed on an agarose gel and visualized using ethidium bromide. The bands as shown are predominantly rRNAs. Equal amounts of an RNA aliquot from the first five fractions were pooled for microarray analysis. RNAs from the other fractions were individually prepared for oligoarray analysis as indicated (see Materials and methods).

**Figure 2:**
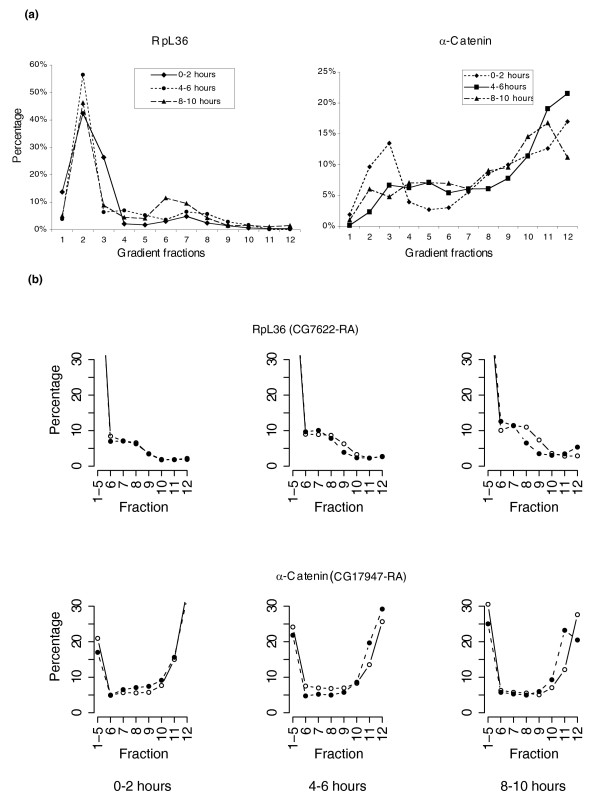
Polysomal profiles of RpL36 mRNA and α-catenin mRNA. **(a) **Quantification of mRNAs encoding RpL36 and α-catenin in individual fractions by real-time PCR. The distribution of each mRNA along the 12 fractions is shown in a percentile scale, which was the average of two independent gradient fractionations. **(b) **Percentile polysomal distribution of RpL36 mRNA and α-catenin mRNA by the measurement of microarrays: x-axis, individual fractions; y-axis, the percentage of a mRNA species in individual fractions. Two sets of microarrays prepared from independent gradient-fractionated RNAs are shown as the open and closed circles individually. The first point is the sum of mRNAs from the first five non-polysomal fractions (fractions 1-5).

We analyzed the translational status of transcripts during early embryogenesis in a genome-wide approach (Figure 1). We chose the three time windows 0-2 hours, 4-6 hours and 8-10 hours after the eggs were laid, to represent the major developmental stages of early *Drosophila *embryogenesis. During the first two hours embryos undergo fertilization, form the preblastoderm and then the syncytial blastoderm. The transition from maternal to zygotic control occurs at about 2 hours post-fertilization. Germ band elongation occurs during 4-6 hours post-egg deposition. Germ band retraction and early organogenesis occur during the 8-10 hours period. We sedimented soluble embryonic extracts through sucrose density gradients to fractionate mRNAs according to their translational status at each developmental period. We analyzed two key indicators of mRNA translation, the density of associated polysomes and the proportion of a given transcript species that is polysomal associated. Measurement of the distributions of mRNAs encoding α-catenin and ribosomal protein L36 (RpL36) in polysomes by microarray hybridization indicated similar profiles to those obtained by quantitative RT-PCR measurements with gene-specific probes (Figure 2a, b). As expected, α-catenin mRNA with a 2,751 nucleotide open reading frame (ORF) sedimented in the high molecular weight fractions 11 or 12, while RpL36 mRNA with a 345 nucleotide ORF peaked in low molecular weight fractions 6 and 7. The rapidly sedimenting complexes containing these mRNAs can be completely abolished by depletion of magnesium with 15 mM EDTA, consistent with the association of these mRNAs with polysomes (data not shown). A predominant amount of RpL36 mRNA in the non-polysomal fraction (fractions 1-5) indicates that a high proportion of RpL36 mRNA was translationally silenced in mRNA-protein (mRNP) complexes.

### Analysis of ribosomal density

**Figure 3:**
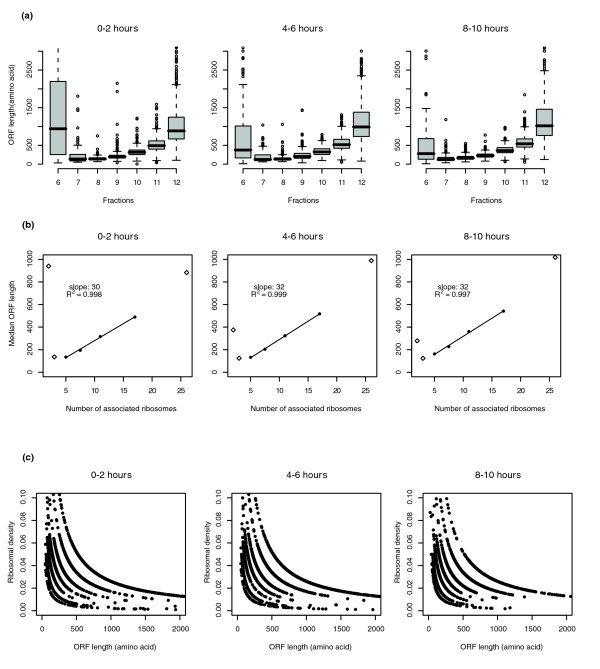
Ribosomal density analysis. **(a) **Boxplots of the ORF length of all the RNA that peaked in each fraction. The plots include only the genes that have a clearly defined EDTA-releasable peak on their polysomal profiles and a signal intensity higher than the median of all the signals at each time point. Thus, 2,237 genes were included at 0-2 hours, 2,282 genes at 4-6 hours and 2,446 genes at 8-10 hours. x-axis, polysomal fractions 6-12; y-axis, transcript ORF length (amino acid). The box stretches from the ORF length at lower 25th percentile to that at the 75th percentile; the median of the ORF length is shown as a line across the box. The vertical dashed lines indicate the ORF length ranges of individual fractions. **(b) **The median ORF length of mRNAs peaking in each fraction versus the assigned number of associated ribosomes in each fraction. The assigned number of associated ribosomes of fractions 6-12 is in the order of 2, 3, 5, 7.5, 11, 17 and 26, respectively. x-axis, the assigned number of associated ribosomes; y-axis, ORF length (amino acids). The slope and the coefficient of determination (R^2^) are shown with each trend line. The data points for fractions 6, 7 and 12 are shown as diamonds, which are not included in the slope calculation. **(c) **Inverse correlation of the ribosomal density and ORF length. x-axis, ORF length (amino acids); y-axis, ribosomal density that is defined as the ratio of the estimated number of associated ribosomes to the ORF length of mRNA (amino acid).

The size of a polysome is determined by the number of associated ribosomes per mRNA transcript and, for a given mRNA, can be estimated from the position of the peak of gradient fractions containing that mRNA. The polysomes with fewer than 10 ribosomes per mRNA are well separated on 20% to 50% sucrose gradients, and the large polysomal complexes (> 10 ribosomes per mRNA) were assigned to fractions 11 and 12 by a logarithmic extrapolation [11]. As expected, we observed that the average ORF length of the mRNA species sedimenting in a given fraction increases from the low molecular weight fractions to the high molecular weight fractions (Figure 3a). The size of polysomes associated with a given mRNA can be affected by changes in the efficiency of ribosomal initiation, elongation and termination. In most cases studied, ribosomal initiation on a transcript is the rate-limiting step, and so the average distance between two adjacent ribosomes on a transcript is mainly determined by ribosomal initiation efficiency. With a same initiation rate, the number of ribosomes per unit length of mRNA, which we call the mRNA ribosomal density, correlates with the length of the ORF of an mRNA [13].

To estimate the average ribosomal density, we used the ratio of the assigned number of associated ribosomes at each fraction to the median ORF length of all the mRNAs sedimenting in that fraction (Figure 3b). It is known that many maternal mRNAs in *Drosophila *early embryos are sequestered in mRNP complexes and sedimentation of these mRNAs is not simply a function of ORF length. Because mRNA-ribosome associations are typically magnesium-dependent, polysome-associated mRNAs can be released from high molecular weight fractions in the presence of EDTA. Therefore, we excluded the mRNAs that cannot be released from high molecular weight fractions by EDTA. However, we found a significant number of mRNAs with long ORFs sedimenting in the disome (2 ribosomes per mRNA) in fraction 6 and trisome (3 ribosomes per mRNA) in fraction 7 (Figure 3a). We cannot distinguish between ribosome-containing mRNP complexes and translating mRNA-ribosomal complexes with the EDTA treatment assay. Furthermore, it is difficult to accurately estimate the number of ribosomes for the extremely long ORFs sedimenting in fraction 12. Therefore, we have less confidence in the accuracy of the average ribosomal density for the mRNAs sedimenting in fractions 6, 7 and 12 and excluded these three fractions from ribosomal density estimation. The average number of associated ribosomes in fractions 8 to 11 shows a linear relationship with the median ORF length of mRNAs (Figure 3b). Therefore, the average ratio of ORF lengths to the number of their associated ribosomes is the slope of this trend line. We estimate the average spacing of ribosomes on the majority of transcripts in *Drosophila *embryos to be about 30-32 amino acid codons, giving an average ribosomal density of about 90-100 nucleotides per ribosome. Most mRNAs have a ribosomal density in the range of 25-40 codons per ribosome at all three developmental stages.

Arava *et al*. [11] reported an unexpected inverse correlation in *S*. *cerevisiae*, namely that ribosomal density decreases with increasing ORF length. In a subsequent study, they concluded that the less frequent initiation of translation of mRNAs with longer ORF is responsible for the observed inverse correlation between the ORF length and ribosomal density [14]. When we analyzed our data using the methods described in Arava *et al*. [11], we also found an inverse correlation (Figure 3c). However, the inverse correlation may simply result from the wide range of ORF lengths seen for the transcripts sedimenting in a given fraction, while one single number of associated ribosomes is assigned to all these transcripts. Instead, we found a fairly stable ratio between the median ORF length of transcripts sedimenting within a given fraction and the assigned number of associated ribosomes to that fraction (Figure 2b). Because the exact number of associated ribosomes to individual transcripts is not clearly defined by the polysome gradient analysis used here and by Arava *et al*., the assertion that ribosomal density decreases with increasing ORF length may not be completely justified.

The consistency of the average ribosomal density during the three developmental time periods we examined indicates that translational regulation of polysome size during early embryogenesis is exerted in a gene-specific manner, rather than at a general level. Based on available ORF lengths in Flybase, about 1.5% of transcripts have a ribosomal density lower than 200 nucleotides per ribosome at each time period and most of them stay in low-density polysomes throughout all three development periods. These mRNAs might have a lower ribosomal initiation rate due to modulation by certain cis-regulatory elements in the mRNA UTRs. However, we could not identify by computational methods any consensus features among those transcripts with a significantly low ribosomal density. Although long UTRs are likely to contain cis-regulatory RNA elements, we did not observe any correlation between the length of the ribosomal density and 5' UTR or 3' UTR length. This may result from both the limitation of computational analysis and the mixture of various cis-RNA regulatory elements within these UTRs. In addition, a prevailing feature of the polysomal profiles of individual transcripts at each of the three time periods examined is that the mRNAs are either sequestered from polysomes or fully loaded with polysomes to an extent correlated with their ORF lengths. This bimodal pattern suggests that most cis-RNA elements in the UTRs are likely to regulate the amount of a transcript associated with polysomes, instead of controlling the ribosome density of a transcript. Furthermore, we did not find significant over-presentation of any particular Gene Ontology (GO) terms among the mRNAs in the lowest 5% density. Our analysis of the 50 mRNAs with highest densities of associated ribosomes revealed that their calculated densities are derived from incorrectly predicted ORF lengths, or a possible cross-hybridization signal from their long alternatively spliced isoforms. These issues make it difficult to pinpoint the highest or lowest ribosomal densities from our analyses. However, these analyses do identify mRNAs whose ORF predictions warrant re-examination.

### Translation activity and ribosomal occupancy during embryonic development

**Figure 4:**
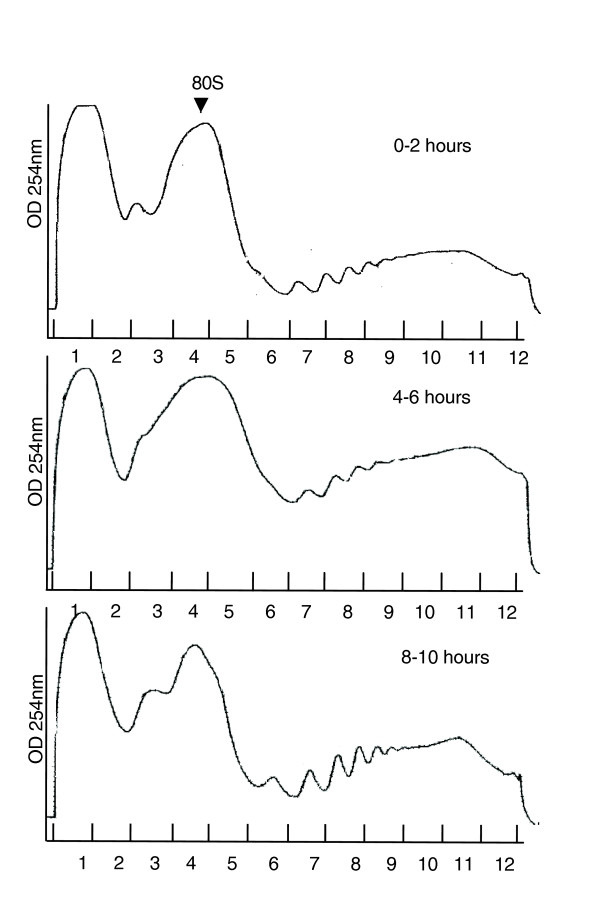
The ultraviolet absorbance profiles at OD 254 nm from 0-2 hour, 4-6 hour and 8-10 hour old embryos.

A key feature of translation status is the proportion of mRNAs associated with polysomes. The relative amounts of free ribosomes (fractions 1-5) to ribosomes engaged in polysomes (fractions 6-12) in the global polysomal profiles are similar at each of the three developmental time periods we examined (Figure 4). Since rRNAs account for more than 95% of total cellular RNAs in most known cell types, and a similar level is seen in cellular RNAs recovered from embryonic lysates, it is likely that rRNA levels or assembled ribosomes remain steady during early embryogenesis [9]. Thus, the number of ribosomes incorporated into polysomal complexes is fairly constant during early development. Furthermore, the appearance of significant amounts of free ribosomes at fractions 3, 4 and 5 also indicates that the availability of ribosomes does not limit the global translation efficiency. To rule out the possibility that the polysomes are arrested at the elongation step, we pulse-labeled *in vitro *translation lysates from 0-2 hour, 4-6 hour and 8-10 hour old embryos with [^35^S]-methionine/cysteine and did not detect any dramatic difference of [^35^S] incorporation (data not shown). Therefore, global translation efficiency appears to be fairly constant throughout early embryogenesis.

**Figure 5:**
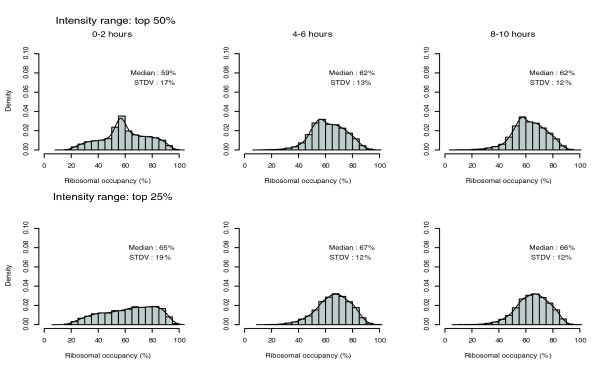
Analysis of ribosomal occupancy. Histograms of ribosomal occupancy, which is defined as the percentage of polysomal associated mRNAs for individual transcript species. The top panels include the probe sets with the top 50% signal intensity among all the probe sets and the bottom panels only include the probe sets with the top 25% intensity. The x-axis is the value of ribosomal occupancy (%). The spacing of rectangles is 5%. The heights of rectangles (y-axis) are densities, defined as density = (number of genes with their value within the 5% range of individual rectangle/total number of genes in the set)/width (5% in these plots). Therefore, the total area of all rectangles equals 1. The lines over the rectangles are the smoothed histograms. STDV, standard deviation.

The percentage of an individual RNA species in the polysomal fractions (fractions 6-12) is defined as the transcript's ribosomal occupancy [11]. The ribosomal occupancies of individual transcripts in each time period covered a wide range, from 20% to 100%, with most mRNA species only partially loaded on polysomes (Figure 5). Thus, the availability of ribosomes and transcripts are excluded as rate-limiting factors for global translational control, and amplitude of translation of individual transcripts is modulated by gene-specific translation factors. The distribution of ribosomal occupancies at 0-2 hours appear more dispersed than that at the later stages, which reflects the diverse mechanisms of translational regulation of specific maternal transcripts (Figure 5).

We classified mRNA species into three statistically defined groups reflecting their translational status: a preferentially translated group, a preferentially untranslated group, and the general group of remaining transcripts. Selected groups of mRNA species were defined by using a logit (see Materials and methods and Additional data file 1). Due to the dispersed distribution of ribosomal occupancy at 0-2 hours, more transcripts were found to be either preferentially translated or preferentially untranslated mRNAs than at the later stages. To understand the biological significance of translational control at each developmental stage, selected groups of mRNAs were clustered by the GO terms of biological process, molecular function and cellular component using the Affymetrix NetAffx analyses tool [15]. We identified the representative significant GO terms among the selected groups, which are listed in Tables 1 and 2 (0-2 hour old embryos), and Tables 3 and 4 (4-6 hour old embryos).

Some nuclear proteins, such as factors with general RNA polymerase II transcription activity (GO0016251) and transcription regulator activity (GO0030528) are the essential components of early zygotic transcription and embryonic pattern formation. It is perhaps unsurprising that most of their mRNAs are associated with polysomes, preferentially synthesizing their protein products in 0-2 hour old embryos (Table 2). At two hours after fertilization, the process of cellularization starts, forming mononucleate blastoderm cells and zygotic transcription begins. The active translation of proteins involved in RNA processing and metabolism in the 4-6 hour old embryos may facilitate the transition to active zygotic transcription. At this stage, nuclear proteins continue to be preferentially translated (Table 4). In contrast, synthesis of ribosomal proteins (rp) is highly inefficient in spite of the high abundance of their transcripts in 0-2 hour old and 4-6 hour old embryos (Tables 1 and 3). The selective silencing of rp-mRNAs during early embryo development is also observed in *Xenopus *in that mRNAs encoding ribosomal proteins are initially in mRNP particles and start to become mobilized to polysomes at a later stage [16], reflecting a need for new ribosomes [17]. A sufficient number of maternal ribosomes are stored in early *Drosophila *embryos before zygotic control begins (Figure 4) and the priority of ribosomal protein synthesis is low. Furthermore, many components involved in macromolecular metabolism, including lipid membrane and protein metabolism, are not observed to be actively engaged in polysomes, and we speculate that their products are also abundantly supplied in the maternal cytoplasm and are not a priority for protein synthesis. Another interesting group of preferentially untranslated mRNAs encode products involved in cell cycle progression (for example, cyclin G and cyclin T) that may also be due to the maternal storage of these products to support the rapid early mitotic cycles. At up to 8-10 hours, when organogenesis starts, we observed no significance in the molecular function or biological process among the selected groups of translated transcripts, which may indicate the complexity of differentiation at this development stage. Sixteen mRNAs encoding proteins involved in RNA binding (for example, *Staufen *and *Smaug*) are not found associated with translating polysomes. These mRNAs are known to play critical roles during oogenesis and early embryogenesis. *In situ *expression staining suggests these transcripts are restricted to germ cells. Due to the rapid degradation of these mRNAs after zygotic transcription begins, it is unclear whether these mRNAs are zygotic or maternal transcripts that are protected in the germ cells.

**Figure 6:**
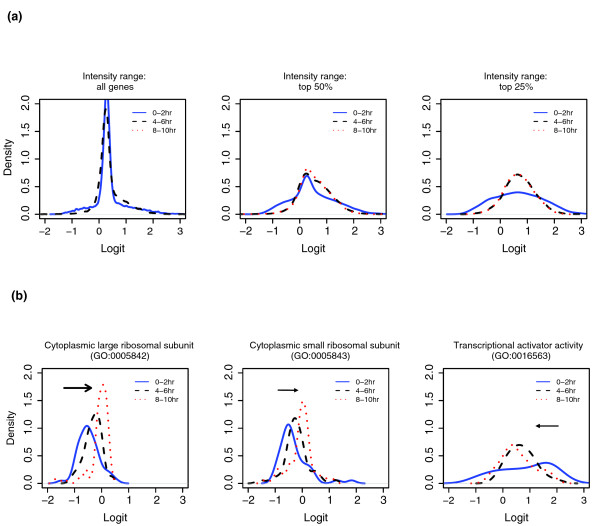
Comparison of polysomal association between 0-2 hour, 4-6 hour and 8-10 hour old embryos. **(a) **Smoothed histograms (density plots) of Logits for individual transcript species from 0-2 hour, 4-6 hour and 8-10 hour old embryos were determined. x-axis, Logit, defined as log {percent(6-12)/percent(1-5)}; y-axis, density of Logit. The left panel includes all the probe sets, the middle panel includes the probe sets with the top 50% signal intensity and the right panel includes only the probe sets with the top 25% intensity. **(b) **Smoothed histograms (density plots) of Logits of the genes encoding cytoplasmic large ribosomal proteins (GO0005842, 56 genes, left), cytoplasmic small ribosomal proteins (GO0005943, 48 genes, middle) and transcription activator (GO0016563, 35 genes, right). The arrows point in the direction of development.

From the GO cluster analyses, we found that mRNAs involved in a common process or cellular components can be specifically co-regulated over the stages of development. Sufficient maternally deposited ribosomes may result in the selective translational repression of rp-mRNAs in early *Drosophila *embryogenesis, and rp-mRNAs gradually move into polysomes when embryos require more ribosomes at the later developmental stages. In comparison to the general polysomal association profile, both the group of large ribosomal proteins (GO0005842) and the group of small ribosomal proteins (GO0005843) were gradually increased from early development (0-2 hour embryos) and finally reached the average level of translational activity at 8-10 hours. We performed a two-sample Kolmogorov-Smirnov test of the null hypothesis of equal distribution to confirm the significance of the polysomal-association increase of rp-mRNAs as groups (GO0005842 and GO0005843) over the three development periods. In contrast, the group of mRNAs with transcription coactivator activity (GO0016563) was preferentially associated with polysomes at 0-2 hours and their association decreased to the average level at 8-10 hours (Figure 6b). There is no significant difference in the overall polysome association of all the probe sets and the probe set including mRNAs with the top 50% abundance among the three time periods (Figure 6a). In contrast, polysome-association of the highly abundant mRNAs (top 25%) in 0-2 hour old embryos is, on average, less than at the later time (Figure 6a, right-hand panel). This suggests that, in general, those abundant maternal transcripts are present in great excess over their requirements for translation.

### Regulation of mRNAs of ribosomal proteins by 5' TOP elements

The rp-mRNAs of *Drosophila *and *Xenopus *embryos always appear either in the mRNPs or fully loaded with ribosomes [17,18]. In *Xenopus*, the translation efficiency of mRNAs encoding several protein components of the translational apparatus, including ribosome proteins, is predominantly dependent on the status of cellular growth. This mode of regulation strictly depends on the 5' terminal location of the oligopyrimidine (5' TOP) tract and sequences immediately downstream of the 5' TOP [16]. A characteristic oligopyrimidine tract starting at a C residue has been found in transcripts encoding ribosomal proteins; the 5' and 3' UTRs of these genes are significantly smaller than the genome average [19]. Ribosomal subunit 40S protein S6 phosphorylation has been implicated in an up-regulation of translation of mRNAs encoding components of the protein synthesis machinery that contain a TOP in their 5' UTR. The concomitant activation of translation of TOP-containing mRNA led to the notion that rpS6 phosphorylation increases the affinity of ribosomes for TOP-containing mRNAs and thus facilitates their initiation [16,20]. S6 knockout mice show decreased growth and cell size [21] and disruption of the *Drosophila *gene encoding S6 kinase leads to small body size and growth rate. Because S6 kinase regulates ribosomal protein production in mammals, loss of *Drosophila *S6 kinase function may have a direct impact on cell growth and proliferation [22]. Therefore, selective translational control of TOP-containing mRNAs might be a translational repression mechanism, which has been evolutionarily conserved in early embryogenesis. However, we could not confirm the existence of the TOP sequence motif in *Drosophila *rp-mRNA 5' UTRs due to lack of the complete 5' UTR sequences of most of these mRNAs.

The selective translational repression of particular mRNA species in response to a reduced cellular need for their protein products might apply to other mRNAs sequestered in 0-2 hour embryos, particularly mRNAs encoding the components of the macromolecule biosynthesis machinery. However, there is no evidence of these mRNAs carrying 5' TOP elements or responding to TOP signaling regulation. Co-regulation of these transcripts implies that translation of these mRNAs is controlled by some shared features, which remain to be defined.

### Coordination of mRNA abundance and translation regulation

At none of the three developmental stages we examined did we find any notable general correlation between polysome-association and mRNA abundance (data not shown). Thus, translation activity is not simply determined by level of transcript accumulation, but more likely reflects dynamic cellular requirements for particular polypeptides. Our study did reveal trends in the relationship between changes in transcript levels and ribosomal occupancies and shows that these trends can vary over the course of development.

**Figure 7:**
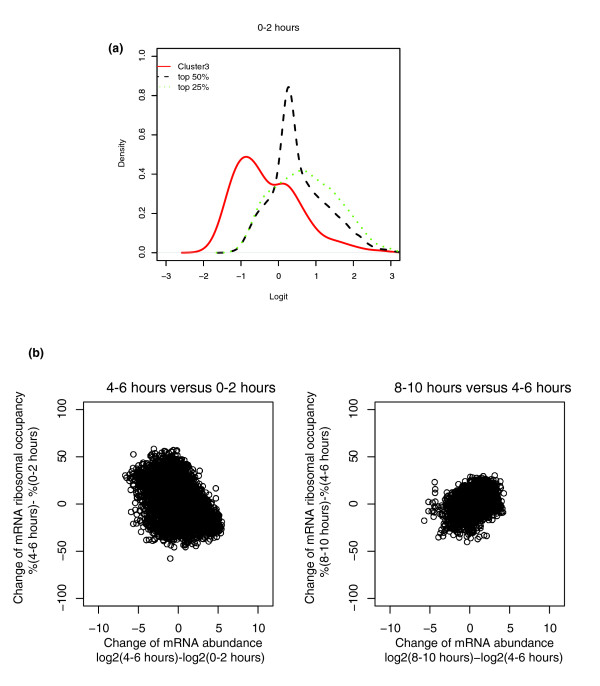
Correlations between change of mRNA abundance and ribosomal occupancy. **(a) **Smoothed histograms (density plots) of Logits of 169 maternal mRNAs (cluster 3 as described by Tomancak *et al*. [23]) and the density plots of the probe sets with the top 50% signal intensity and the probe set with the top 25%. x-axis, Logit; y-axis, density of Logit. **(b) **Scatter plot of change in ribosomal occupancy against change of mRNA abundance over the time course.

A comparison of 4-6 hour and 0-2 hour embryo samples shows that where mRNA levels decrease during very early embryogenesis, there is a tendency for ribosomal occupancies to increase (Figure 7b, left). This change may simply reflect the rapid degradation of excess maternal transcripts after zygotic control begins, and not necessarily any upturn in the level of protein synthesis. For example, a gene cluster including 169 maternal mRNAs whose steady state level quickly drops two hours after egg deposition was identified by Tomancak *et al*. [23]. The average ribosomal occupancy of these 169 mRNAs is much lower than that of the majority of other transcripts at 0-2 hours, whose levels change less between the 0-2 hour stage and the 4-6 hour stage (Figure 7a). It is likely that the general inverse correlation of changes in mRNA abundance and ribosomal loading is simply a consequence of clearing maternal transcripts at the zygotic transition.

On the other hand, comparing ribosomal occupancies in 8-10 hour and 4-6 hour embryos reveals a positive correlation with changes in transcript abundance (Figure 7b, right). This correspondence of elevation of mRNA accumulation with increased ribosomal occupancies indicates some coordination of steady state RNA levels and translation activity; the levels of gene regulation underlying this phenomenon and how directly and generally they are integrated remain questions for future investigation.

**Figure 8:**
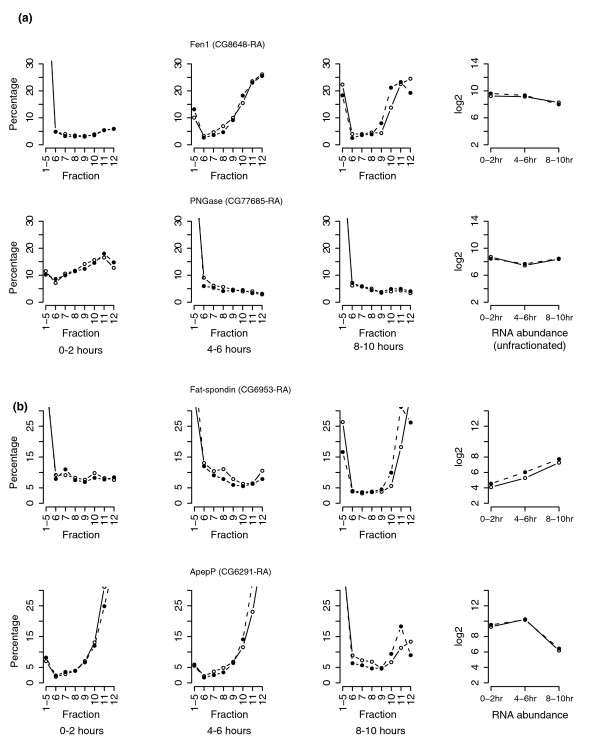
Percentile polysomal profiles of representative mRNAs whose polysomal association is highly regulated during early embryonic development. **(a) **Translational regulation between 0-2 hours and 4-6 hours. Duplicate array experiments prepared from independently fractionated RNA samples are shown individually. The first point is the sum of mRNAs from the first five non-polysomal fractions (fractions 1-5). Fen1 is up-regulated after the first two hours while PNGase is down-regulated. Their relative mRNA abundance is plotted in the far right panel. The abundance is shown as a Log2 scale (y-axis), which is determined by the signal intensity on the GeneChips and normalized as described in Materials and methods. **(b) **Translational regulation between 4-6 hours and 8-10 hours. Fat-spondin is up-regulated between 4-6 hours and 8-10 hours while ApepP is down-regulated. The right panel illustrates the relative mRNA abundance at each time period.

Using a multivariate empirical Bayes (MB) statistic [24], we ranked all the transcripts according to the difference between ribosomal occupancy between 0-2 hours and 4-6 hours, and between 4-6 hours and 8-10 hours (Additional data file 2). The higher a gene's rank, the more dramatic is the observed change in its polysomal profile. The percentile polysomal profiles of representative mRNAs with high rankings are shown in Figure 8. Expression of these genes is likely to be translationally regulated. We were not able to identify any significantly shared features of the primary sequences among mRNAs with high MB rankings, although they are expected to be modulated in response to certain common development signals.

### Polysomal profiles of localized transcripts in *Drosophila *embryos

For the spatially localized mRNAs that have been studied, such as *nanos *mRNA, their spatial localization and translation control are often closely linked, with translation being repressed during mRNA translocation and activated on reaching its destination [3,25,26]. Until this study, biochemical analysis of ribosomal association to estimate mRNA translation status has been completed for only few *Drosophila *mRNAs. We evaluated the polysome association profiles of several known localized mRNAs during embryogenesis.

**Figure 9:**
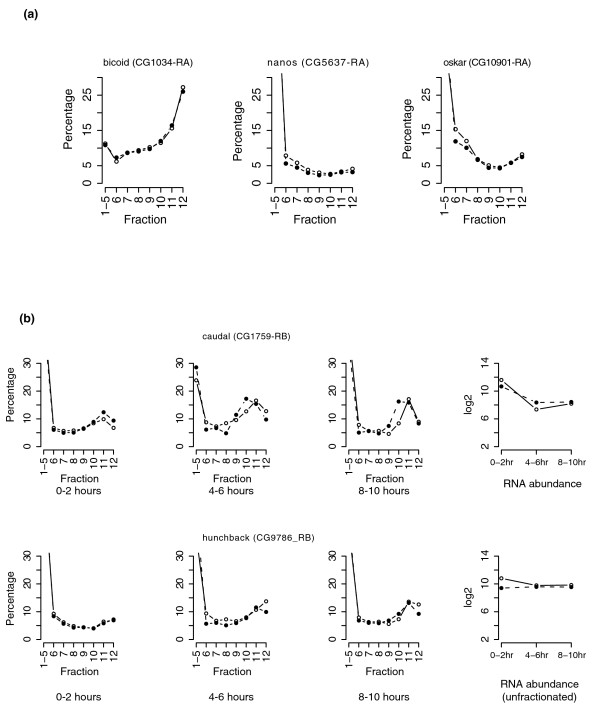
Percentile polysomal profiles of several known subcellularly localized transcripts. **(a) **Percentile polysomal profiles of *nanos*, *oskar *and *bicoid *from 0-2 hour old embryos. **(b) **Percentile polysomal profiles of caudal mRNAs and hunchback mRNAs at 0-2 hours, 4-6 hours and 8-10 hours. Two sets of microarrays prepared from independent gradient-fractionated RNAs are shown as the open and closed circles individually. The right panel illustrates their relative mRNA abundance (y-axis) at each time period (x-axis).

Our observation that only a small proportion of *nanos *(5% in fraction 12) is associated with polysomes is consistent with the published estimate; it has been reported that about 4% of *nanos *mRNA is localized to the posterior and actively translated [27] (Figure 9a). However, Clark *et al*. [27] found a comigration of 53% of *nanos *mRNA associated with polysomes and suggested, based on their study, that *nanos *mRNA translation is regulated in a novel manner. We do not understand the basis of this difference between our observations and those of Clark *et al*.

Consistent with immunocytological observations that translation of *hunchback *transcripts and *caudal *transcripts is regionally repressed in the early embryo [3,26], the experiments presented here indicate that only a small proportion of these transcripts are associated with polysomes (Figure 9b). The unique polysomal profiles of maternal transcripts, for example, the transcripts of *nanos*, *oskar *and *bicoid*, suggest the existence of multiple mechanisms controlling gene-specific translation of mRNAs during early pattern formation.

## Conclusion

Translational control is critical for early *Drosophila *embryogenesis and we used a genomic approach to illustrate the translation profile of transcripts during this developmental period. The raw microarray data analysis tables and polysomal profiles of individual transcripts are available online at the Berkeley *Drosophila *Genome Project (BDGP) homepage [28]. The diversity of the polysomal profiles of maternal transcripts (0-2 hour old embryos) and later zygotic transcripts indicates multiple complex mechanisms that modulate individual gene expression, but also co-regulate the genes involved in same biological processes. The identification of consensus regulatory elements within such co-regulated mRNAs, as well as trans-acting factors that recognize them will be a fruitful area of future study.

## Materials and methods

### Synchronization of embryo collections

Canton S embryos were collected in 2-hour intervals and aged to generate animals 0-2, 4-6 and 8-10 hours old. To confirm that the embryos were collected at the desired developmental stages, we examined the morphology of a small aliquot of the synchronized embryos as described previously [23]. In addition, we also validated the synchronization by comparing the variation of RNA abundance of representative mRNAs over the three time periods with the previous microarray measurements performed by Tomancak *et al*. [23] (data not shown). The embryos were then dechorionated and transferred to Eppendorf tubes.

### Preparation of RNA samples

Unfractionated RNA was prepared by homogenization of dechorionated embryos with a motorized plastic pestle in RNAwiz solution (Ambion, Austin, TX 78744-1832), followed by chloroform extraction and ethanol precipitation. To prepare the polysome-associated RNAs, the dechorionated embryos were first incubated with 0.1 mg/ml cycloheximide in PBS for 10 minutes on ice, then homogenized with a motorized plastic pellet pestle in a lysis buffer (20 mM Tris-HCl, pH 7.4, 140 mM KCl, 5 mM MgCl_2_, 0.5 mM DTT, 1% Triton X-100, 0.1 mg/ml cycloheximide, 1 mg/ml heparin, 50 unit/ml RNasin) and incubated for 10 minutes on ice. The debris were removed by centrifugation at 12,000 × g for 10 minutes at 4°C, and supernatants were loaded onto 20% to 50% sucrose gradients with the same extraction buffer without Triton X-100. The extracts were sedimented at 35 k rpm for 160 minutes in a SW41 rotor at 4°C. Twelve fractions were collected from the tops of the gradients using an ISCO fraction collection system. RNAs were precipitated from each fraction with guanidine hydrochloride and ethanol followed by a second precipitation in 1.5 M LiCl at -20°C overnight. The RNA precipitate was washed with 70% ethanol and resuspended in an equal volume of Tris-HCl buffer (1 mM Tris-HCl, pH 8.0). Purified RNAs from individual fractions were quantified with a spectrophotometer and visualized on formaldehyde agarose gels [27,29].

EDTA-treated embryos were lysed in an EDTA extraction buffer (20 mM Tris, pH 7.4, 140 mM KCl, 15 mM EDTA, 0.5 mM DTT, 1% Triton X-100, 0.1 mg/ml cycloheximide, 1 mg/ml heparin, 50 unit/ml RNasin) and sedimented through 20% to 50% gradients prepared with the same EDTA lysis buffer, but without Triton X-100.

Although most cytoskeleton-associated and endoplasmic reticulum-associated RNAs as well as ribosomes are released into the soluble extract under this buffer condition (X Qin, unpublished data), it is possible that some mRNAs are sequestered in insoluble complexes and excluded from polysomal gradient analysis. RNAs potentially in the insoluble debris were not characterized in this study.

### Quantitative PCR analysis

Either unfractionated total RNA or equal proportions of RNA from each polysomal fraction were reverse transcribed into cDNAs with a High Capacity cDNA Archive kit (Applied Biosystems, Inc. Foster City, CA 94404). Gene-specific TaqMan^® ^probes were designed and manufactured through Assay-by-design (Applied Biosystems). Equal proportions of cDNA samples mixed with TaqMan^® ^Universal PCR Master Mix and gene-specific TaqMan^® ^probes were quantified in a 96-well plate on ABI PRISM^® ^7000 Sequence Detection Systems as described by Applied Biosystems.

### Microarray hybridization and data analysis

RNAs from the first five gradient fractions were pooled. An equal volume of the pooled RNAs and RNAs from the remaining seven fractions was used for cRNA labeling. Thus, the pooled RNA sample used for array labeling was the average amount of RNA from the first five fractions and each RNA sample contained at least 10 μg of RNAs. cRNA was hybridized to a GeneChip *Drosophila *Genome 2.0 Array using standard protocols. Thus, we collected eight GeneChip array scans of each polysomal gradient and the success of the experiments was determined by the reproducibility of the two independent replicates. Similarly, we prepared four pools of RNA from the first five fractions, from fractions 6 and 7, from fractions 8 and 9 and from fractions 10, 11 and 12. These four RNA samples were used for microarray analysis of the EDTA treated samples. Total RNA (20 μg) from unfractionated cell lysates at each time point was used for one-step labeling and GeneChip hybridization. Gene expression measures were normalized and computed using the robust multichip average (RMA) method described in [30] and implemented in the Bioconductor R package. Statistical analyses were all performed with the open-source software R, version 2.2.0 and Bioconductor 1.7 packages [31]. The following R packages were used mainly; Affy (version 1.8.1), limma (version 2.2.0) and *Drosophila *2 (version 1.10.0) [32].

Moderated t-statistics were used to determine whether a transcript is released from polysomes by EDTA filtering [33]. If the amount of a particular gene's mRNA in the first fraction of EDTA-treated profiles is higher than that in the first fraction of non-EDTA-treated profiles, this transcript is releasable by EDTA since the materials dissociated by EDTA are expected to sediment at the pooled non-polysomal fraction. The false discovery rate (FDR) was controlled at *p *= 0.05. All data for such genes were removed for further analyses. Among all the 18,952 probes on the *Drosophila *Genome 2.0 GeneChips, 16,513 genes at 0-2 hours, 16,519 genes at 4-6 hours and 14,593 genes at 8-10 hours were left for further data analysis. In addition, we excluded mRNAs with low signal intensity to exclude the background noise as well as possible signal saturation of those mRNAs with extremely high intensity as described in individual analyses.

### Peak selection and ribosomal association assignment

To determine the peak fraction of each mRNA for ribosomal density estimation, we used m as the measure for selecting genes with a sharp peak in their polysomal profiles. We first averaged the two normalized replicates of polysomal gradients on the logarithmic scale. Next, we removed the genes whose transcripts were not releasable by EDTA and whose intensities were below the median of all the probe sets. Then, we calculated m as the following:

m = % [peak fraction] - average% [adjacent two fractions], if peak fraction is not 6 or 12

m = % [peak fraction] - % [fraction 7] if peak = 6

m = % [peak fraction] - % [fraction 11] if peak = 12

The 3,000 genes with highest m values were expected to have a distinct peak and were selected to estimate the ribosomal density at each time interval.

The number of ribosomes per transcript in fractions 6-10 was obtained directly from the peaks in the average of multiple OD254 profiles (Figure 3a). For fractions 11 and 12, which lacked single ribosome resolution, the number of ribosomes per transcript was estimated by a logarithmic extrapolation from the clearly defined peaks as described by Arava *et al*. [11]. The assigned number of associated ribosomes for fractions 6-12 is in the order of 2, 3, 5, 7.5, 11, 17 and 26, respectively. The R^2 ^value of the logarithmic curve over the defined region is 0.9983 (data not shown).

### Cluster transcripts with their translational activity

We used two parameters to describe the polysomal profiles: the logarithmic ratio of the polysomal fractions to non-polysomal fractions (Logit):

Logit = log [percent(fraction 6-12)/percent(fraction 1-5)]

and the standard deviation (SD) of the expression levels among all the gradient fractions.

The preferentially translated mRNAs are expected to have a high Logit and a high SD, while unpreferentially translated mRNAs have low Logit and low SD. We examined the distribution of Logit and SD of each time point (data not shown) and decided to use a cutoff of Logit > 1.5 and SD > 0.5 to define actively translated genes, while those with log ratio < -0.15 and SD < 1 are defined to be translationally inactive genes. Any other genes not included in these two clusters were defined to be in the general translation group.

### Gene Ontology analyses

Clustered genes were analyzed using the NetAffx Gene Ontology Mining Tool provided by Affymetrix [34]. The goal of GO analysis was to find statistically overrepresented GO terms within a group of genes [35]. Also of interest is comparison of the distribution of a statistic such as the Logit among genes associated with a certain GO term. The GOstats package of R was used to get GO-filtered data. The distributions of Logit for a set of genes such as those associated with a given GO term can be compared across time points. For this purpose, a two-sample Kolmogorov-Smirnov test of the null hypothesis of equal distribution was performed.

## Additional data files

The following additional data are available with the online version of this paper. Additional data file 1 lists the genes that were classified by Logit into the preferentially translated group and the preferentially untranslated group at each development stage. Additional data file 2 includes the MB statistic ranking lists of the top 500 genes, showing the most significant changes of their ribosomal occupancy between the development periods.

## Supplementary Material

Additional data file 1 Genes that were classified by Logit into the preferentially translated group and the preferentially untranslated group at each development stage Click here for file

Additional data file 2 MB statistic ranking lists of the top 500 genes, showing the most significant changes of their ribosomal occupancy between the development periods Click here for file

## Figures and Tables

**Table 1:** GO terms for mRNA transcripts preferentially excluded from polysomes at 0-2 hours(1,404 genes)

Term	GO	No. of genes in *D. melanogaster *database	No. of genes in the selected group	*p *value
Biological process (918 genes*)				
Biosynthesis	GO0009058	996	189	2.47E-16
Primary metabolism	GO0044238	5,032	638	3.85E-07
Macromolecule biosynthesis	GO0009059	579	136	5.55E-22
Protein biosynthesis	GO0006412	548	130	1.48E-21
Translation	GO0043037	214	51	4.21E-09
Amino acid activation	GO0043038	66	22	1.36E-08
Lipid metabolism	GO0006629	542	82	4.14E-03
Membrane lipid biosynthesis	GO0046467	33	12	5.46E-06
Response to endogenous stimulus	GO0009719	150	34	9.39E-06
Response to external stimulus	GO0009605	714	34	8.83E-09
Cell communication	GO0007154	1,928	159	1.60E-06
Cell cycle	GO0007049	520	92	1.90E-06
				
Molecular function (933 genes*)				
Structural constituent of ribosome	GO0003735	205	70	4.08E-26
Ligase activity	GO0016874	345	71	1.56E-08
Receptor activity	GO0004872	666	30	1.31E-08
Nucleic acid binding	GO0003676	1,988	314	6.86E-14
				
Cellular component (637 genes*)				
Cytoplasm	GO0005737	1,695	304	1.60E-23
Ribosomes	GO0005840	206	71	5.53E-26
Cytosolic large ribosomal subunit	GO0005842	56	30	3.18E-23
Cytosolic small ribosomal subunint	GO0005843	48	21	1.97E-12

*Number of the probe sets in the selected group with an available annotation as biological process, molecular function or cellular component.

**Table 2:** GO terms for mRNA transcipts preferentially associated with polysomes at 0-2 hours (906 genes)

Term	GO	No. of genes in *D. melanogaster *database	No. of genes in the selected group	*p *value
Biological process (535 genes*)				
Development	GO0007275	1,992	181	2.01E-07
Pattern specification	GO0007389	318	38	8.90E-05
Embryonic pattern specification	GO0009880	148	22	4.45E-05
Blastoderm segmentation	GO0007350	118	19	2.86E-05
Regulation of biological process	GO0050789	1,681	143	3.76E-04
Regulation of cellular process	GO0050794	1,561	139	3.92E-05
Gametogenesis	GO0007276	574	64	4.82E-06
				
Molecular function (539 genes*)				
Transcription regulator activity	GO0030528	971	88	5.05E-04
RNA pol II transcription activity	GO0003702	362	45	2.43E-06
General RNA pol II transcription activity	GO0016251	110	37	1.94E-13
Transcriptional activator activity	GO0016563	35	11	1.78E-09
Transcription cofactor activity	GO0003712	86	16	4.42E-06
Transcriptional coactivator activity	GO0003713	24	8	8.58E-08
DNA binding	GO0003677	947	85	1.12E-04
RNA binding	GO0003723	438	50	1.57E-05
				
Cellular component (392 genes*)				
Nucleus	GO0005634	1,793	324	2.21E-18
Nucleoplasm	Go0005654	249	42	6.97E-10
Transcription factor complexes	GO0005667	92	19	3.16E-07
DNA-directed RNA polymerase II, holoenzyme	GO0016591	91	21	1.97E-09
Actin filament	GO0005884	26	14	1.08E-20

*Number of the probe sets in the selected group with an available annotation as biological process, molecular function or cellular component.

**Table 3:** GO terms for mRNA transcripts preferentially excluded from polysomes at 4-6 hours (621 genes)

Term	GO	No. of genes in *D. melanogaster *database	No. of genes in the selected group	*p *value
Biological process (333 genes*)				
Macromolecule biosynthesis	GO0009059	579	88	1.53E-44
Protein biosynthesis	GO006412	548	87	9.10E-04
				
Cellular component (252 genes*)				
Eukaryotic 43S pre-initiation complex	GO0016282	70	32	3.22E-62
Eukaryotic translation initiation, factor 3 complex	GO0005852	15	8	1.05E-19
Cytosolic large ribosomal subunit	GO0005842	56	33	6.68E-86
Cytosolic small ribosomal subunint	GO0005843	48	23	1.35E-47

*Number of the probe sets in the selected group with an available annotation as biological process, molecular function or cellular component.

**Table 4:** GO terms for mRNA transcipts preferentially associated with polysomes at 4-6 hours (536 genes)

Term	GO	No. of genes in *D. melanogaster *database	No. of genes in the selected group	*p *value
Biological process (319 genes*)				
Nucleobase, nucleoside, nucleotide and nucleic acid metabolism	GO0006139	2,008	138	3.96E-15
RNA metabolism	GO0016070	394	46	4.27E-16
RNA processing	GO0006396	271	28	3.33E-08
Transcription initiation from pol II promoter	GO0006367	92	13	4.14E-07
				
Cellular component (216 genes*)				
Nucleus	GO0005634	1,793	114	5.77E-11
DNA-directed RNA polymerase II, holoenzyme	GO0016591	91	17	2.01E-13
Spliceosome complexes	GO0005681	111	16	7.56E-09
Small nuclear ribonucleoprotein complex	GO0030532	63	13	5.12E-12
Proteasome regulatory particle	GO0005838	24	11	1.90E-26

*Number of the probe sets in the selected group with an available annotation as biological process, molecular function or cellular component.
